# Image features and clinical analysis of retroperitoneal pelvic schwannoma: a case report

**DOI:** 10.1186/s12883-024-03715-y

**Published:** 2024-07-03

**Authors:** Xining Wu, Hua Meng, Qingbo Fan, Zhenhong Qi, Weidong Pan

**Affiliations:** 1 Department of Ultrasonography, Peking Union Medical College Hospital, Chinese Academy of Medical Sciences and Peking Union Medical College, Shuaifuyuan NO.1, Dongcheng District, Beijing, China; 2 Department of Obstetrics and Gynecology, Peking Union Medical College Hospital, Chinese Academy of Medical Sciences and Peking Union Medical College, Shuaifuyuan NO.1, Dongcheng District, Beijing, China; 3 Department of Radiology, Peking Union Medical College Hospital, Chinese Academy of Medical Sciences and Peking Union Medical College, Shuaifuyuan NO.1, Dongcheng District, Beijing, China

**Keywords:** Femoral, Pelvic, Retroperitoneal, Schwannoma

## Abstract

**Background:**

Schwannomas are benign usually encapsulated nerve sheath tumors derived from the Schwann cells, and affecting single or multiple nerves. The tumors commonly arise from the cranial nerves as acoustic neurinomas but they are extremely rare in the pelvis and the retroperitoneal area. Retroperitoneal pelvic schwannomas often present with non-specific symptoms leading to misdiagnosis and prolonged morbidity.

**Case presentation:**

We report the case of a 59-year-old woman presenting with a feeling of heaviness in the lower abdomen who was found to have a retroperitoneal pelvic schwannoma originating from the right femoral nerve. She had a history of two resections of peripheral schwannomas at four different sites of limbs. After conducting magnetic resonance imaging, this pelvic schwannoma was misdiagnosed as a gynecological malignancy. The tumor was successfully removed by laparoscopic surgery. Pathological analysis of the mass revealed a benign schwannoma of the femoral nerve sheath with demonstrating strong, diffuse positivity for S-100 protein.

**Conclusions:**

Although retroperitoneal pelvic schwannoma is rare, it should be considered in the differential diagnosis of pelvic masses, especially in patients with a history of neurogenic mass or the presence of neurogenic mass elsewhere.

## Background

Schwannomas (neurilemmomas), are encapsulated nerve sheath tumors derived from the Schwann cells, and affecting single or multiple nerves [1]. Most schwannomas are benign and grow slowly. Schwannomas can arise in peripheral, cranial, or visceral nerves at any anatomic site of the human body [2].These tumors commonly arise from the cranial nerves as acoustic neurinomas, but are extremely rare in the pelvis and the retroperitoneal area [3]. Retroperitoneal schwannomas often present with non-specific symptoms leading to misdiagnosis and prolonged morbidity. Retroperitoneal pelvic schwannomas are easily confused with gynecological tumors. Herein, we report a case of multiple schwannomas, most likely schwannomatosis, in which the retroperitoneal pelvic schwannoma was located in the right femoral nerve, misdiagnosed as gynecological malignancy by preoperative imaging.

Due to numbness in the left middle finger and ring finger, the patient underwent resection of a solid tumor in the left axilla 27 years ago. The postoperative pathology showed that it was a schwannoma originating from the median nerve. 16 years ago, the patient developed right palm pain, right calf and sole pain, and multiple soft tissue masses in the right upper and lower extremities could be palpated on physical examination. Surgery confirmed that these tumors were all schwannomas, originating from the right ulnar, sciatic, and peroneal nerves, respectively. In the past 5 years, the patient complained of occasional paroxysmal radiating pain in the right inguinal area and the right leg, accompanied by a feeling of bulging in the lower abdomen, and no obvious abnormality in the motor and sensory function of the lower limbs.

## Case presentation

**Fig. 1:**
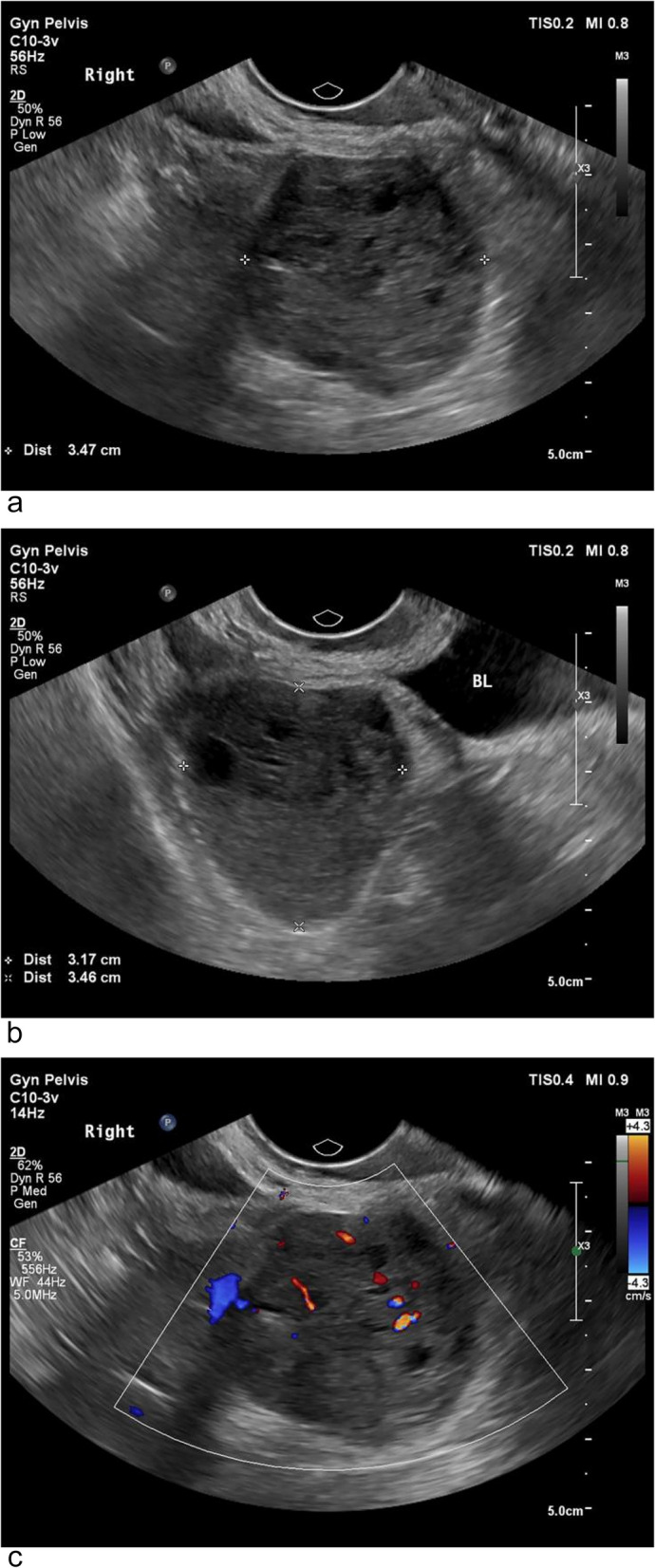
The transvaginal ultrasound at diagnosis. **a** and **b** A well-defined, hypoechoic mass is in the right adnexa, adjacent to the bladder. **c** Color Doppler image shows the mass contains internal flow. *BL *bladder

**Fig. 2:**
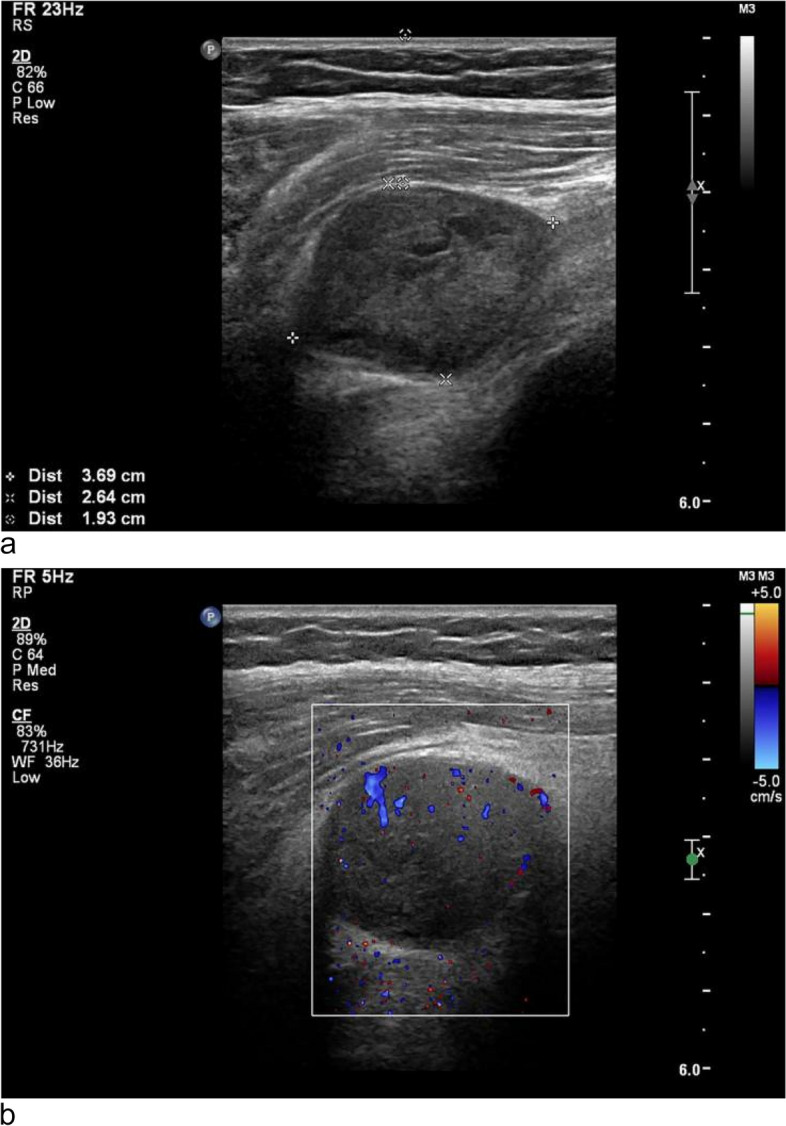
Ultrasound shows a solid mass in the deep muscle of right thigh suspiciously originating from the nerve. **a** The mass is located 1.9 cm away from the thigh surface. **b** Doppler flow can be detected in the mass

**Fig. 3:**
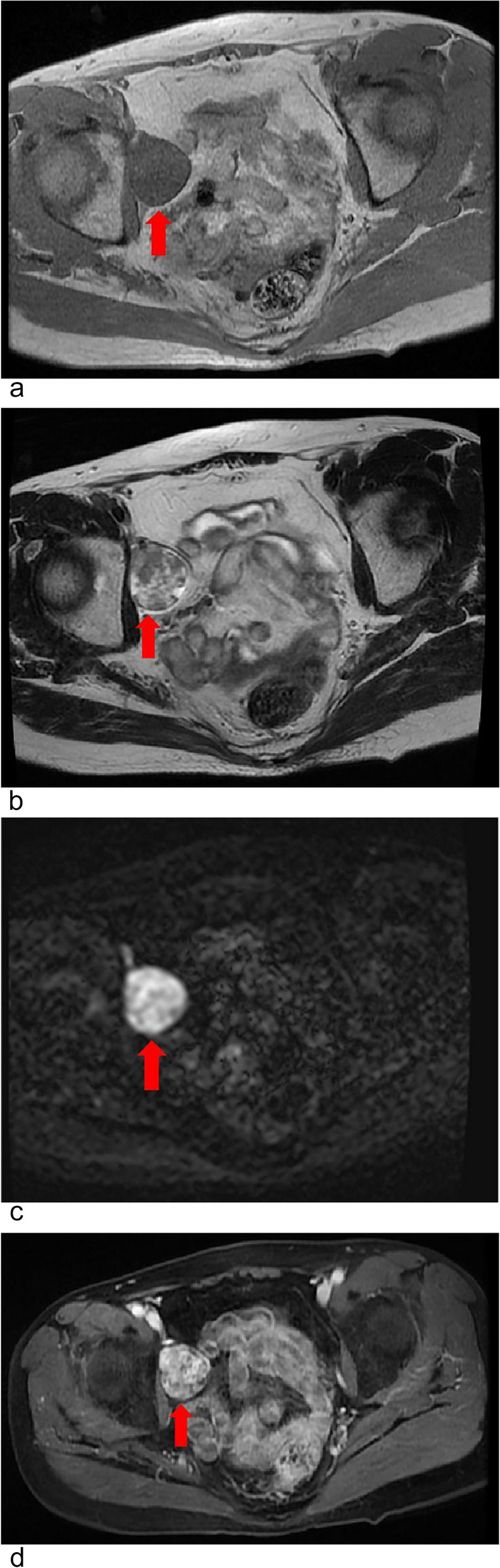
The MRI reveals a round mass in the pelvis (arrow). **a** Hypointense on T1. **b** Hyperintense but heterogenous on T2. **c** Hyperintense on diffusion-weighted imaging. **d** The solid part of the mass with obvious contrast-enhancement. *MRI *magnetic resonance imaging

**Fig. 4:**
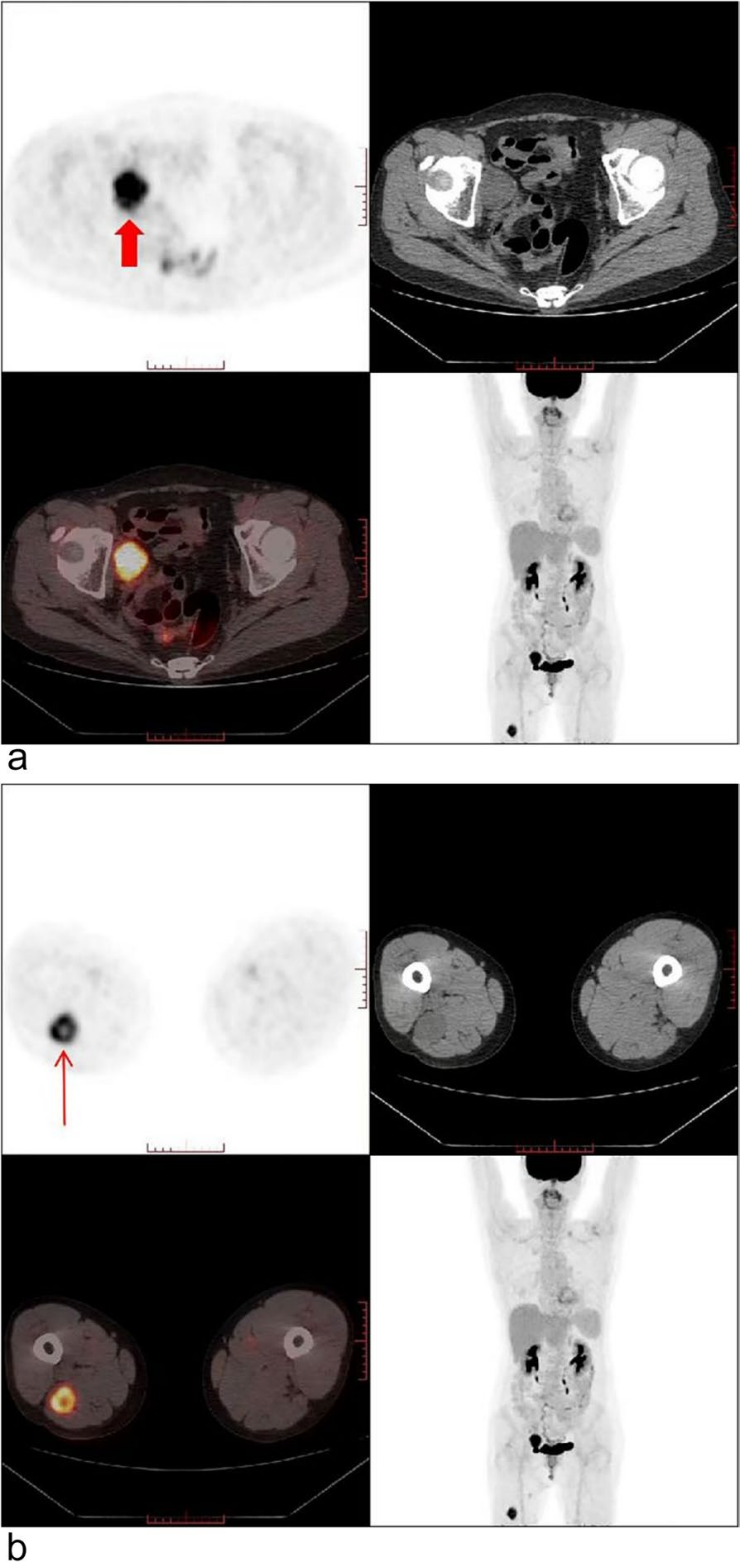
The trunk PET/CT examination confirm the presence of the pelvic mass and the lesion in the right thigh muscles. **a** and **b** Both the pelvic mass (thick arrow) and the mass in the right thigh (thin arrow) show high tracer uptake. *PET/CT *positron emission tomography and computed tomography

A 59-year-old postmenopausal woman, gravida 2 para 1, obtained a pelvic ultrasound examination at a local hospital 1 year ago. Ultrasound showed multiple uterine fibroids and a cystic-solid mass in the right adnexal area with a size of 3.5 × 3.4 × 3.0 cm. The patient underwent laparoscopic resection of the entire uterus and both adnexa at the same hospital, and no tumor was found in the bilateral adnexal areas during the operation. Histopathology of both ovaries was normal. Half a year after the operation, the patient came to the gynecology out-patient department of our hospital complaining a sense of heaviness in the lower abdomen. During the physical examination, a solid mass was touched in the right pelvis with obvious tenderness. The transvaginal ultrasound scan showed a 3.5 × 3.2 × 3.5 cm solid-based mass in the right adnexa, adjacent to the bladder. The mass was well-defined, hypoechoic with multiple small anechoic cystic areas, vascularized on Doppler (Fig. 1). During the examination, the patient described a palpable nodule on the inner of her right thigh. Ultrasound showed a 3.9 × 2.6 cm solid mass in the deep muscle of right thigh, which was considered as a neurogenic tumor (Fig. 2). The level of CA125, AFP and CEA were normal. The pelvic magnetic resonance imaging (MRI) revealed a round mass on the right side of the pelvis, hypointense on T1, hyperintense but heterogenous on T2, hyperintense on diffusion-weighted imaging, and the solid part of the mass with obvious contrast-enhancement (Fig. 3). The appearance of the lesion on MRI was suggestive of a malignant tumor of the right ovary. After that, the patient underwent a trunk ^18^ F-fluorodeoxyglucose positron emission tomography and computed tomography (FDG-PET/CT) examination, which confirmed the presence of the pelvic mass with increased tracer uptake, and a metabolic enhancement of lesion was also seen in the right thigh muscles, with 2.7 cm, both considered malignant lesions (Fig. 4).

After consultation, the surgeon considered that this pelvic mass was likely to be a schwannoma of retroperitoneal origin combining the patient's medical history and clinical symptoms. On August 2021, the patient was hospitalized and opted for surgical removal of the pelvic mass. Laparoscopic exploration showed smooth pelvic floor and lateral pelvic wall peritoneum, and no obvious protrusions and space-occupying lesions were found. The right retroperitoneum was opened along the stump of the infundibulopelvic ligament, clearly showing the lateral ureter and iliac vessels. The obturator fossa was explored, and no significant lesions were found.

**Fig. 5:**
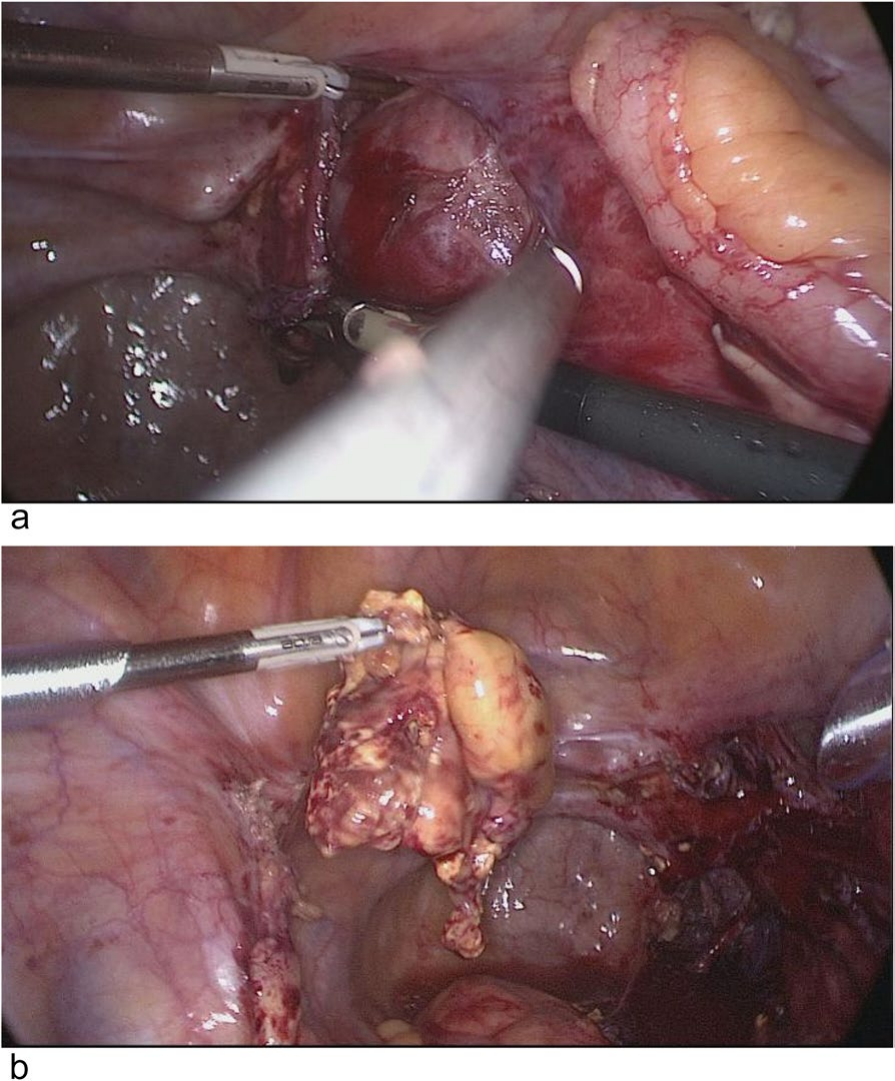
Laparoscopic procedure. **a** Encapsulated solid mass in the retroperitoneal pelvis. **b** The mass was removed intact

**Fig. 6:**
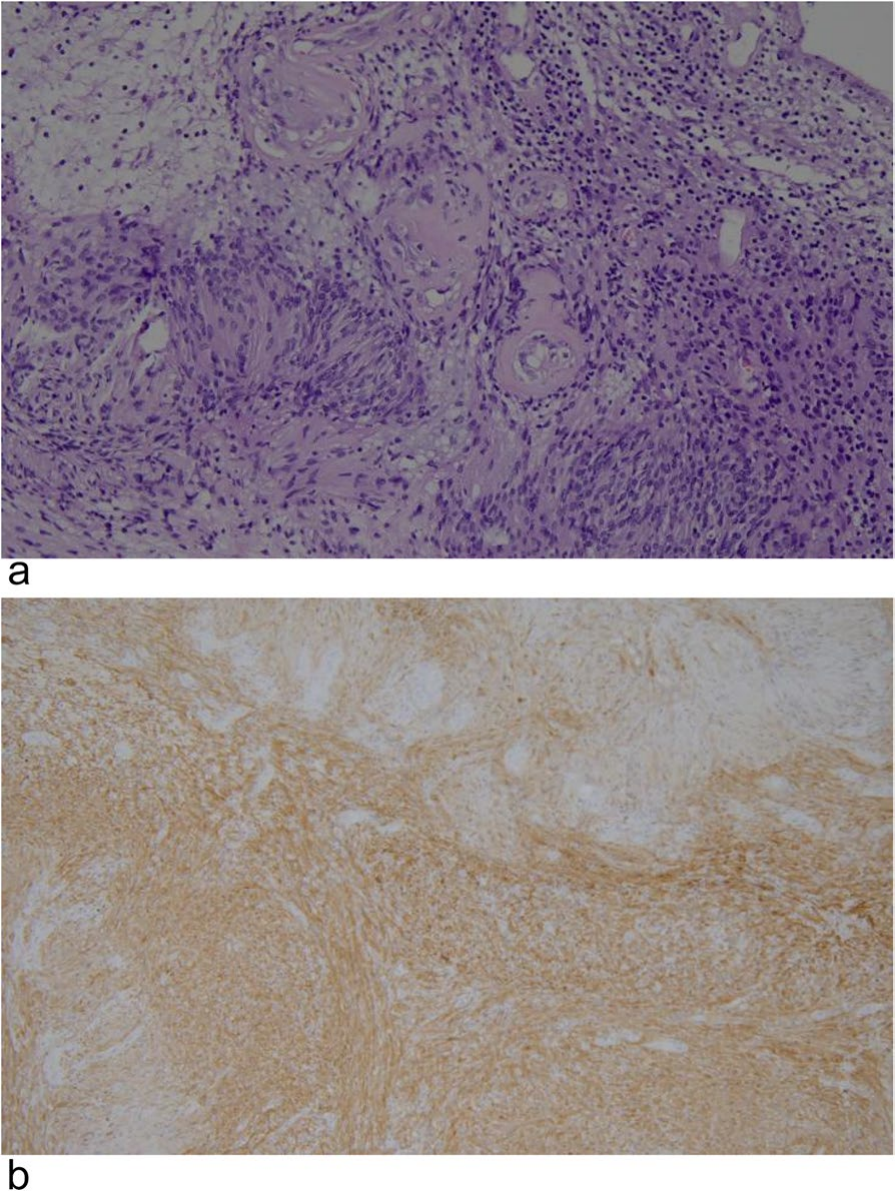
Histopathologic examination of the benign schwannoma. **a** × 200. **b** Strong immunohistochemical positivity for S-100 protein

The gynecologist checked the MRI and PET/CT images again, and finally determined that the mass was located in the retroperitoneum of the pelvic floor on the right side of the bladder. Cutting the peritoneum on the surface of the mass, a smooth and encapsulated solid mass was visible (Fig. 5), attached to the right femoral nerve surface. The right quadriceps muscle was contracted and the right hip and knee joints were straightened when the mass was touched. The tumor well dissected from the surface of the femoral nerve and totally removed with no neural damage. Macroscopically, the mass was cystic and solid with yellow bean dregs-like contents. Finally, the tumor was diagnosed as a benign retroperitoneal schwannoma, and histopathological morphology showed on standard coloration a spindle-shaped proliferation of cells without cyto-nuclear atypia, with numerous hyalinized vessels. On immunohistochemistry, the whole schwannoma cellular population demonstrated strong, diffuse positivity for S-100 protein (Fig. 6). After surgery, the patient recovered smoothly and left the hospital 7 days later. The patient had no numbness, pain or abnormal movement of the lower limbs during follow-up.

## Discussion and conclusions

To our knowledge, this is the first case of a retroperitoneal pelvic schwannoma following a history of resection of multiple peripheral schwannomas of the extremities, and the present mass in the right thigh is also considered as a schwannoma. The presence of multiple schwannomas is associated with neurofibromatosis (NF). There are three types of NF: type 1 (NF1) accounting for 96% of all cases, type 2 (NF2) in 3%, and schwannomatosis in < 1% [4, 5]. Schwannomatosis is characterized by multiple schwannomas of the peripheral nervous system in the absence of vestibular schwannomas [6]. This patient has no family history of neurofibromas, no NF1- or NF2-related stigmata on physical examination, and no clinical manifestations of hearing impairment. Although she did not have a cranial MRI examination, schwannomatosis is the most likely diagnosis.

Schwannomas are rarely found in the retroperitoneal region (less than 0.5% of cases) [2]. When located in pelvis, they can be misdiagnosed as uterine myoma [7] or adnexal masses [8, 9]. Retroperitoneal schwannomas are slow-growing tumours and do not cause many symptoms until they have attained a large size. They may cause some symptoms secondary to pressure on adjacent nerves, vessels and organs, such as vague pain in the lower back, abdomen and pelvis. Most patients have no specific clinical symptoms, and the preoperative diagnosis rate is low, which often leads to insufficient preoperative preparation and a passive state during surgery.

Preoperative diagnosis of pelvic and retroperitoneal schwannomas is difficult as there are no specific imaging findings. A review of 82 retroperitoneal schwannomas revealed that only 15.9% were identified preoperatively by ultrasound, CT, or MRI [10]. MRI is the optimal imaging tool for these kinds of tumors [11], since it is able also to show its possible origin, vascularization, and invasiveness, and can even definitively diagnose pelvic schwannomas [3]. MRI also helps to clarify the relationship with neighboring pelvic organs (sacrum, rectum, and bladder), which is an important consideration for the surgical procedure [12], but misdiagnosis is not uncommon. In addition, MRI is often used to check the abdominal and pelvic cavity, but multiple schwannomas such as soft tissues of the limbs can not be simultaneously explored. Even experienced radiologists cannot comprehensively consider giving an accurate diagnosis. Some scholars suggested that a whole-body MRI examination should be uesed to exclude multiple schwannomas [13]. This patient had a clear history of uterine and oophorectomy surgery, and the MRI report still suggested an ovarian-derived malignant mass, possibly due to the heterogeneity of schwannoma and internal cystic changes similar to the appearance of an ovarian malignant tumor.

Ultrasonography has an important role in detecting pelvic tumors. However, pelvic schwannoma has no specific ultrasound signs and usually presents as a homogeneous hypoechoic, well-defined mass, with internal cystic changes and calcification, which is easily confused with tumors derived from uterus and ovary. With technological advances in the quality of ultrasound resolution, pelvic nerves can be visualized, a novel ultrasound imaging, ‘pelveoneurosonography’ has the potential to evaluate pelvic nerves and track the source of the tumor [13]. However, the specific effects need to be further verified by prospective studies. For patients with multiple schwannomas, ultrasound may have advantages. It can easily detect suspicious masses in other parts than the pelvis and abdominal cavity, especially in the limbs. When the probe presses the tumor and symptoms of nerve irritation occur during the examination, it is helpful to confirm the diagnosis of neurogenic tumors. This patient underwent multiple limbs schwannoma resections, and this time the right thigh mass was also diagnosed as a neurogenic tumor by ultrasound. If all these information can be taken into account, the pelvic mass may be obtained closer to the correct diagnosis before surgery. There is no consensus on the recommendation of ultrasound-guided tumor biopsy, given the difficulties of interpretation, or the risk of bleeding and infection [12, 13]. However, gynecologic ultrasound is also an optimal modality for the surveillance of retroperitoneal pelvic schwannoma, especially if the patient is asymptomatic [13].

In this case, PET/CT was performed for suspected gynecological malignancy, showing an increased accumulation of FDG in the pelvic mass and the right thigh mass, all of which were misdiagnosed as malignant lesions. According to the previous report, the large accumulation of FDG in schwannomas might result from the potential ability of schwann cells to transport glucose for axonal repolarization in the peripheral nerve system [14]; however, a consensus has not been reached on the precise mechanism of FDG accumulation in schwannomas. In our experience, when there are multiple schwannomas, PET/CT examination may be unreliable, and it is easy to misdiagnose multiple lesions as metastases. Therefore, the application of PET/CT is not recommended.

Surgical resection of the entire schwannoma is the optimal treatment. Care to avoid injury to adjacent structures and to accomplish a complete excision of the mass is essential for the safe performance of this surgery. Resection without the correct technique can cause permanent neurological deficits and pain and recurrence in up to 54% of patients [15]. Schwannomas are solitary, well-circumscribed, encapsulated tumors and do not invade local tissues. Due to these characteristics they are easily dissected from adjacent tissues. The laparoscopic approach is an effective and minimally invasive option for the resection of retroperitoneal pelvic schwannoma. However, for large retroperitoneal schwannoma, laparotomy should also be considered in order to ensure complete resection. Because patients with schwannomatosis often may undergo multiple operations at different sites during their lifetime, an interdisciplinary approach is the optimal strategy to manage benign schwannoma [15]. This patient of our study will undergo elective surgery on the right thigh mass.

To sum up, although retroperitoneal pelvic schwannoma is rare, it should be considered in the differential diagnosis of pelvic masses, especially in patients with a history of neurogenic mass or the presence of neurogenic mass elsewhere. Accurate diagnosis of retroperitoneal pelvic schwannoma ramains a challenge. Management and diagnostic experience in the course of this case, although limited, emphasizes the importance of a complete preoperative history collection, physical examination, and detailed imaging evaluation, which can improve the outcome of surgical treatment. The final diagnosis is established at histopathological examination.

## Data Availability

The datasets used during the current study are available from the corresponding author on reasonable request.

## Abbreviations

MRI: Magnetic resonance imaging
CT: Computed tomography
FDG: F-fluorodeoxyglucose
PET: Positron emission tomography
NF: Neurofibromatosis
